# One hypervirulent clone, sequence type 283, accounts for a large proportion of invasive *Streptococcus agalactiae* isolated from humans and diseased tilapia in Southeast Asia

**DOI:** 10.1371/journal.pntd.0007421

**Published:** 2019-06-27

**Authors:** Timothy Barkham, Ruth N. Zadoks, Mohammad Noor Amal Azmai, Stephen Baker, Vu Thi Ngoc Bich, Victoria Chalker, Man Ling Chau, David Dance, Rama Narayana Deepak, H. Rogier van Doorn, Ramona A. Gutierrez, Mark A. Holmes, Lan Nguyen Phu Huong, Tse Hsien Koh, Elisabete Martins, Kurosh Mehershahi, Paul Newton, Lee Ching Ng, Nguyen Ngoc Phuoc, Ornuma Sangwichian, Pongpun Sawatwong, Uraiwan Surin, Thean Yen Tan, Wen Ying Tang, Nguyen Vu Thuy, Paul Turner, Manivanh Vongsouvath, Defeng Zhang, Toni Whistler, Swaine L. Chen

**Affiliations:** 1 Department of Laboratory Medicine, Tan Tock Seng Hospital, Singapore; 2 Institute of Biodiversity Animal Health and Comparative Medicine, College of Medical Veterinary and Life Sciences, University of Glasgow, Glasgow, United Kingdom; 3 Department of Biology, Faculty of Science, and Laboratory of Marine Biotechnology, Institute of Bioscience, Universiti Putra Malaysia, Selangor, Malaysia; 4 Oxford University Clinical Research Unit, Ho Chi Minh City, Vietnam; 5 Centre for Tropical Medicine, Oxford University Clinical Research Unit, Hanoi, Vietnam; 6 Public Health England, Colindale, London, United Kingdom; 7 Environmental Health Institute, National Environment Agency, Singapore; 8 National Centre for Food Science, Singapore Food Agency, Singapore; 9 Lao-Oxford-Mahosot Hospital-Wellcome Trust Research Unit, Mahosot Hospital, Vientiane, Lao People’s Democratic Republic; 10 Centre for Tropical Medicine and Global Health, Nuffield Department of Medicine, University of Oxford, Oxford, United Kingdom; 11 Faculty of Infectious and Tropical Diseases, London School of Hygiene and Tropical Medicine, London, United Kingdom; 12 Department of Laboratory Medicine, Khoo Teck Puat Hospital, Singapore; 13 Oxford University Clinical Research Unit, Hanoi, Vietnam; 14 Nuffield Department of Clinical Medicine, Centre for Tropical Medicine and Global Health, University of Oxford, United Kingdom; 15 National Centre for Infectious Diseases, Singapore; 16 Department of Veterinary Medicine, University of Cambridge, Cambridge, United Kingdom; 17 Department of Microbiology, Singapore General Hospital, Singapore; 18 Instituto de Microbiologia, Instituto de Medicina Molecular, Faculdade de Medicina, Universidade de Lisboa, Lisboa, Portugal; 19 Department of Medicine, Yong Loo Lin School of Medicine, National University of Singapore, Singapore; 20 Faculty of Fisheries, University of Agriculture and Forestry, Hue University, Hue City, Vietnam; 21 Thailand Ministry of Public Health (MOPH)-US Centers for Disease Control and Prevention Collaboration (TUC), Nonthaburi, Thailand; 22 Nakhon Phanom General Hospital, Nakhon Phanom Provincial Health Office, Nakhon Phanom, Thailand; 23 Department of Laboratory Medicine, Changi General Hospital, Singapore; 24 Molecular Biology Laboratory, Tan Tock Seng Hospital, Singapore; 25 National Hospital for Obstetrics & Gynaecology, Hanoi, Vietnam; 26 Cambodia-Oxford Medical Research Unit, Angkor Hospital for Children, Siem Reap, Cambodia; 27 Key Laboratory of Tropical & Subtropical Fishery Resource Application & Cultivation, Ministry of Agriculture and Rural Affairs, Pearl River Fisheries Research Institute, Chinese Academy of Fishery Sciences, Guangzhou, People’s Republic of China; 28 Division of Global Health Protection, Centers for Disease Control and Prevention, Atlanta, Georgia, United States of America; 29 Infectious Diseases Group, Genome Institute of Singapore, Singapore; University of Texas Medical Branch, UNITED STATES

## Abstract

**Background:**

In 2015, Singapore had the first and only reported foodborne outbreak of invasive disease caused by the group B *Streptococcus* (GBS; *Streptococcus agalactiae*). Disease, predominantly septic arthritis and meningitis, was associated with sequence type (ST)283, acquired from eating raw farmed freshwater fish. Although GBS sepsis is well-described in neonates and older adults with co-morbidities, this outbreak affected non-pregnant and younger adults with fewer co-morbidities, suggesting greater virulence. Before 2015 ST283 had only been reported from twenty humans in Hong Kong and two in France, and from one fish in Thailand. We hypothesised that ST283 was causing region-wide infection in Southeast Asia.

**Methodology/Principal findings:**

We performed a literature review, whole genome sequencing on 145 GBS isolates collected from six Southeast Asian countries, and phylogenetic analysis on 7,468 GBS sequences including 227 variants of ST283 from humans and animals. Although almost absent outside Asia, ST283 was found in all invasive Asian collections analysed, from 1995 to 2017. It accounted for 29/38 (76%) human isolates in Lao PDR, 102/139 (73%) in Thailand, 4/13 (31%) in Vietnam, and 167/739 (23%) in Singapore. ST283 and its variants were found in 62/62 (100%) tilapia from 14 outbreak sites in Malaysia and Vietnam, in seven fish species in Singapore markets, and a diseased frog in China.

**Conclusions:**

GBS ST283 is widespread in Southeast Asia, where it accounts for a large proportion of bacteraemic GBS, and causes disease and economic loss in aquaculture. If human ST283 is fishborne, as in the Singapore outbreak, then GBS sepsis in Thailand and Lao PDR is predominantly a foodborne disease. However, whether transmission is from aquaculture to humans, or *vice versa*, or involves an unidentified reservoir remains unknown. Creation of cross-border collaborations in human and animal health are needed to complete the epidemiological picture.

## Author summary

An outbreak due to a bacterium called *Streptococccus agalactiae* in Singapore in 2015 was caused by a clone called ST283, and was associated with consumption of raw freshwater-fish. It was considered unique as it was the only reported foodborne outbreak of this bacterium. Our new data show that invasive ST283 disease is far from unique. ST283 has been causing disease in humans and farmed fish in SE Asian countries for decades. Reports of ST283 are almost absent outside Asia. We suspect that human ST283 is fishborne in other Asian countries, as it was in Singapore, but we haven’t looked at this yet. We don’t know where ST283 originally came from; it may have been transmitted from humans to fish, or come from another animal. More studies are needed to determine ST283’s geographical extent and burden of disease, as well as its origin, how it is transmitted, and what enables it to be so aggressive. We may then be able to interrupt transmission, to the benefit of fish, farmers, and the general public.

## Introduction

In 2015, there was an unprecedented outbreak of invasive disease due to the group B *Streptococcus* (GBS; *Streptococcus agalactiae*) in Singapore. Disease, including septic arthritis and meningitis, was associated with a GBS belonging to serotype III, subtype 4 (serotype III-4), and multilocus sequence type (MLST) 283 (ST283) [1]. Human disease was associated with consumption of raw, farmed, freshwater fish: an official public advisory was issued, and bacteraemia rates promptly fell [2, 3]. Whereas GBS sepsis in neonates, post-partum adults, and older adults with co-morbidities is well known, the ST283 outbreak was different as it affected non-pregnant, younger adults with fewer co-morbidities [1], suggesting greater virulence. Although GBS colonisation has previously been associated with fish consumption [4], and the origin of GBS in late onset neonatal disease, while uncertain, may be enteric [5, 6], this was the first report of an invasive GBS outbreak associated with foodborne transmission.

Previous reports of ST283 were limited to 20 cases in adults in Hong Kong between 1993 and 2003 [7], two cases in a survey of 119 osteoarticular GBS infections in France between 2002 and 2007 [8], and an infected tilapia (*Oreochromis* sp.) amongst samples collected in Thailand between 2000 and 2010 [9]. Southeast (SE) Asia was, however, under-represented in the literature, with MLST data reported for only a handful of human GBS from SE Asia amongst thousands in global studies.

We hypothesised that fish-borne ST283 might be a regional problem, causing disease in multiple SE Asian countries. As a prelude to studying transmission, we searched for ST283 and its variants, collectively called clonal complex 283 (CC283), in GBS collections from humans and aquaculture. Our aim was achieved, as we verified the wide prevalence of ST283 in Southeast Asia, though whether human disease is fish-borne outside Singapore remains an open question.

## Methods

The study was approved by the Institutional Review Board of Tan Tock Seng Hospital (TTSH), Singapore, NHG DSRB 2016/00202.

### Literature search

We identified articles in English with searches of Medline, PubMed, and references from articles, with the terms “*Streptococcus agalactiae*” OR “ST283” AND “meningitis” OR “invasive”, up to December 2017.

### GBS collections

**Fig 1:**
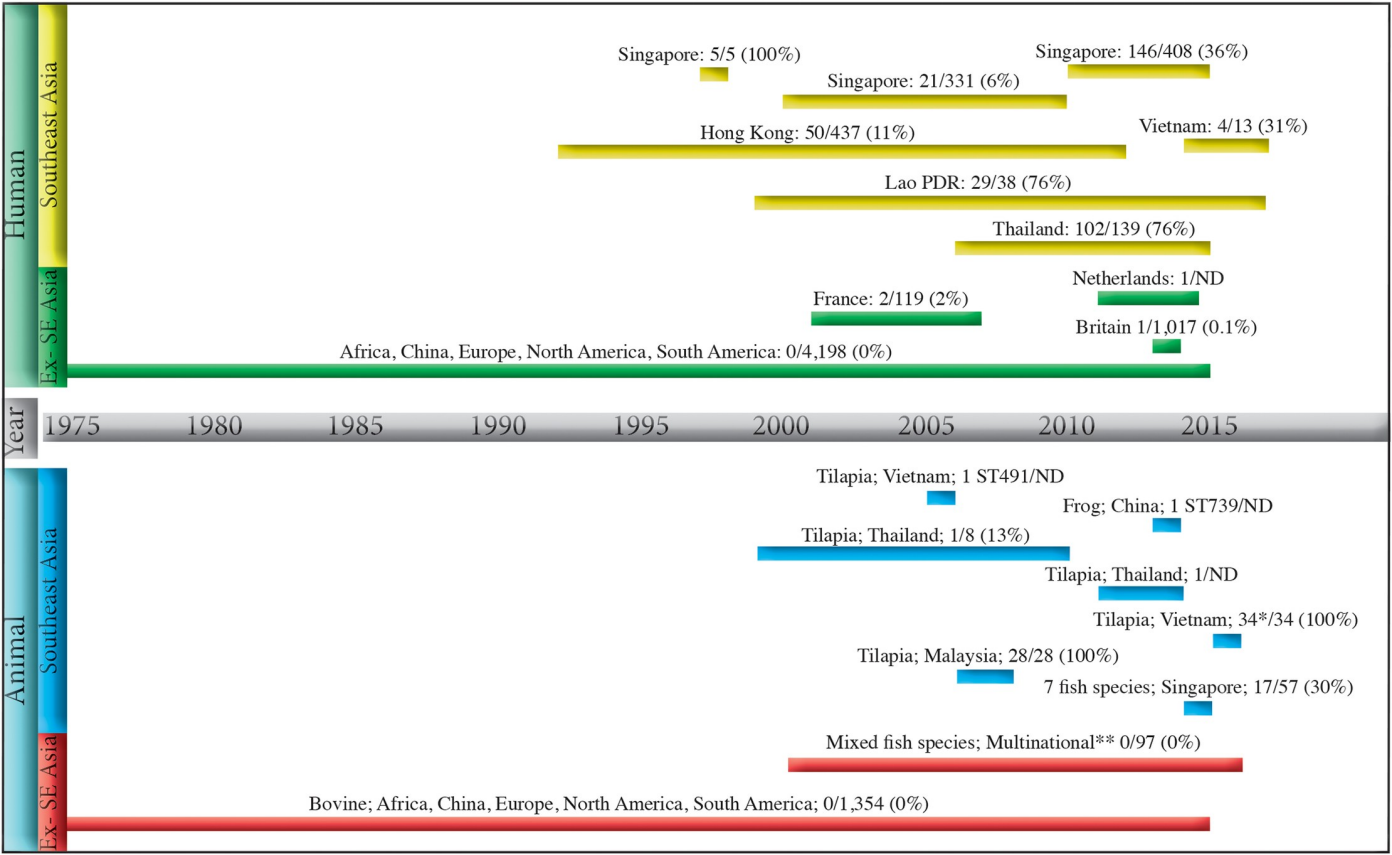
Prevalence of clonal complex 283 in human and animal collections of group B *Streptococcus* showing their host, geographic origin, and period of collection. This figure represents our new data, as well as the literature on *Streptococcus agalactiae* (GBS) that includes multi locus sequencing typing data up to December 2017. The vertical bars on the left indicate human or animal origin and the geographical region, Southeast Asia (SEA) or outside SEA (Ex- SE Asia), where collections of GBS originated. The horizontal bars delineate the time period of each collection of GBS, with reference to the central time bar; associated text shows the host, the country of origin, and number of ST283/all GBS in each collection, except where other STs are shown. The figure shows the lack of reports, from humans and animals, in SEA compared with outside SEA, both in terms of time periods and absolute numbers of GBS studied. It also shows that GBS CC283 is rare outside SEA, with only four human and no animal CC283 reported, despite the large number of GBS studied. In contrast, CC283 is prevalent in all human and animal GBS collections from SEA. *One of these 34 from a tilapia in Vietnam is ST1311, a double locus variant of ST283. ** Australia, Ghana, South America, North America, Israel and Kuwait. Abbreviation: ND = not determined.

Institutions, selected through personal contacts and recommendations, were invited to contribute GBS and/or datasets. There were no specified criteria other than the availability of isolates or DNA from invasive GBS, or GBS from high vaginal swabs, over time, with metadata if possible. Invasive disease was defined as isolation of GBS from normally sterile sites. Indications for testing patients and accompanying characterisation data were not standardised or complete; rates of meningitis, osteoarthritis, and endocarditis may therefore be greater than recorded. Country-specific regulations resulted in some contributors sending GBS isolates to Singapore for processing while others contributed extracted DNA, whole genome sequencing (WGS) data, or other data sets. GBS were screened with a ST283-specific PCR (Lezhava A., Sarma S., Chen S. and Barkham T.M.S. A method for the detection of Group B Streptococcus. (2017) PCT Patent Application PCT/SG2017/050579). This PCR has been evaluated against WGS on over 660 invasive GBS representing 27 MLST types collected over 18 years in Singapore and found to be 100% sensitive and 99.8% specific. GBS identification, determination of serotype and WGS were performed as previously described [1]. All new sequencing data were deposited in GenBank under BioProject PRJNA293392. We expressed ST283 prevalence as simple proportions of all GBS in each collection (Fig 1).

### Human GBS

#### Singapore

Invasive bacterial isolates are routinely saved: 331 invasive GBS isolates from 2001 to 2010 were retrieved from freezers in TTSH and Changi General Hospital. Data and sequences for 408 invasive GBS ST283 isolated from 2011–2015 were publicly available [1]. Sequences of five GBS, isolated in Singapore General Hospital in 1998 from cases of meningitis, are now available at GenBank accessions SRR6282417-SRR6282421 (under BioProject PRJNA417692).

#### Thailand

139 invasive GBS isolates were available from population-based surveillance for blood stream infections conducted in all 20 hospitals in Nakhon Phanom and Sa Kaeo provinces from 2007–2015; these include paediatric patients. GBS isolates from the isolate repository were screened with the ST283 PCR described above. A selection of positive isolates were subjected to WGS on the MiSeq platform using the Nextera XT DNA Library Prep Kits and the MiSeq Reagent Kit v3. In addition, sequences of seven GBS ST283 from invasive human cases in Bangkok in 2015 were publicly available [1].

#### Lao PDR

This set comprised 38 GBS, available as isolates or as DNA extracts, obtained from blood cultures and CSF samples at the Microbiology Laboratory of Mahosot Hospital, Vientiane, Lao PDR between 2000–2017 during studies of the aetiology of fever and central nervous system infections.

#### Vietnam

13 invasive GBS isolated between 2015 and 2017 were retrieved from the Hospital for Tropical Diseases (HTD), Ho Chi Minh City (HCMC). HTD is a 550-bed hospital that serves as a main primary and secondary facility for the surrounding local population in HCMC and a tertiary referral center for infectious diseases for the southern provinces of Vietnam. Nearly 70% of HTD admissions live in HCMC, with the remainder residing in the surrounding provinces. Neonates, patients without infectious diseases, including those with surgical requirements, tuberculosis, cancer, primary hematological disorders or immunosuppression (other than HIV) are referred to other hospitals within HCMC. HIV-infected children are often referred to local paediatric hospitals. Blood cultures were performed for patients in whom an infection was suspected on the basis of a fever (>38°C) or who had evidence of sepsis on the basis of the presence of two or more of the following features: fever (>38°C) or low temperature (<36°C); tachycardia (exact level according to age); tachypnea (exact level according to age); an elevated white cell count (>12,000 cells/mm3) or depressed white cell count (<4,000 cells/mm3). There was no systematic change in the application of these criteria during the time course of the study. All data originating from consecutive patients admitted to the hospital who had a blood culture performed for suspected bloodstream infection between 2015 and 2017 were included. In addition, 38 GBS representing the most recent high vaginal swab GBS isolates from women with colpitis, three of whom were pregnant, were collected from outpatients at the National Hospital for Obstetrics & Gynaecology (Phu San Hospital), Hanoi, between September 2016 and May 2017.

#### Cambodia

DNA from eleven GBS isolated from skin and umbilical swabs collected from children presenting to Angkor Hospital for Children, a non-governmental paediatric referral hospital in Siem Reap, between 2012 and 2016 were included.

#### Britain

WGS data were available from 1,017 invasive human GBS submitted to the Respiratory and Vaccine Preventable Bacteria Reference Unit, Public Health England (PHE), as part of the British Society for Antimicrobial Chemotherapy Resistance Surveillance Project over a one year period bridging 2014 and 2015.

### Piscine GBS

#### Singapore

Sequences of GBS ST283 isolated from fish in 2015 were publicly available [1, 10].

#### Malaysia

We sequenced 28 GBS, isolated between 2007 and 2008, from brain, eye or kidney samples from 28 tilapia (*Oreochromis* sp.) from farms suffering streptococcosis outbreaks. Ten of these 28 tilapia did not show any external or internal signs of disease. The fish were obtained from nine farms separated by 20–250 km in Kedah and Terengganu states, Peninsular Malaysia.

#### Vietnam

We sequenced 34 GBS isolated in 2016 from the brains of sick fish from five farms in the Mekong River running through An Giang Province and Can Tho District. GBS was isolated directly from the brains of red or black tilapia (*Oreochromis* sp. and *O*. *niloticus*) that showed abnormal behaviour (erratic swimming pattern) or “pop eye” (exophthalmus), both of which are recognized clinical signs of streptococcosis.

#### Other datasets

Other GBS MLST datasets were contributed by collaborators. All whole genome data sets annotated as *S*. *agalactiae* in GenBank deposited as assemblies (as of June 07, 2017) and in the SRA database (as of November 10, 2017) were downloaded.

#### Genomic methods

We performed Illumina sequencing on 145 CC283 GBS strains. All new sequencing data was deposited in GenBank under BioProject PRJNA293392. All assembled genomes (as of June 7, 2017) and short read data sets (as of November 10, 2017) annotated as *S*. *agalactiae* in GenBank were also downloaded; this provided another 82 CC283 isolates, for a total of 227 CC283 isolates amongst 7,468 GBS. All primary sequence analysis was performed by the Genome Institute of Singapore Efficient Rapid Microbial Sequencing (GERMS) platform (https://www.a-star.edu.sg/gis/Our-Science/Technology-Platforms/GERMS). Reference-based analyses were performed using the SG-M1 genome [11] as the reference. FASTQ files were mapped using bwa (version 0.7.10) [12]; indel realignment and single nucleotide polymorphism (SNP) calling were performed using Lofreq* (version 2.1.2) with default parameters [13]. SNP positions for assembled genomes were inferred by using nucmer and show-snps from the MUMmer package (version 3.23) [14]. MLST and resistance gene predictions were made using SRST2 0.2.0 [15] for Illumina sequenced strains or using a custom BLASTN [16] based script for fully assembled reference sequences, using the recommended MLST database (http://pubmlst.org/sagalactiae/) [17] and the ARG-ANNOT resistance gene database [18] included with SRST2.

#### Recombination analysis

**Fig 2:**
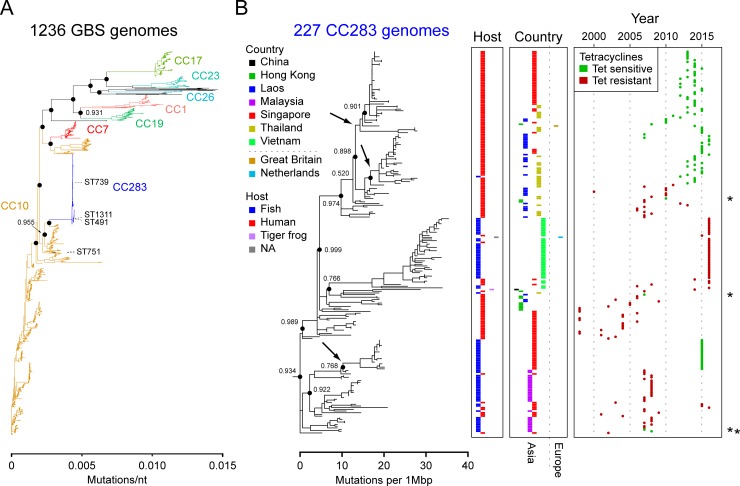
Phylogenetic analysis of group B *Streptococcus* (GBS) with emphasis on clonal complex (CC) 283 genomes. (A) Approximately maximum-likelihood phylogenetic tree of 1,236 GBS strains. The ST283 isolate SG-M1 was used as a reference sequence. The scale bar is shown on the x-axis, in mutations/nucleotide. The bootstrap values for selected branches (supporting the difference between different CCs) are indicated by black circles; all are 1.000 except where indicated. Major clonal complexes are indicated with coloured branches and a matching coloured label. The clade containing all CC283 isolates is highlighted in blue. All ST283 variants discussed in the text (ST491, ST739, ST751, and ST1311) are highlighted with black branches and an adjacent label in black text. (B) Approximately maximum-likelihood phylogenetic tree of 227 GBS CC283 genomes. Reconstructed genome sequences (based on the SG-M1 reference sequence) of the isolates indicated as CC283 in (A) were used, after excluding redundant isolates, defined as identical sequences from the same site, based on SNP calls. Bootstrap support is indicated for selected branches by black circles. Arrows show predicted events resulting in loss of tetracycline resistance. (C) For each CC283 isolate, the metadata are indicated at the same horizontal position (i.e. directly to the right of the phylogenetic tree tip) as in panel B. Host and country are represented by coloured rectangles, as indicated in the legend. Different values for Host and Country are further offset horizontally for clarity. Asterisks to the right of the Year box indicate isolates for which individual loss of tetracycline resistance appears to have occurred.

From an initial neighbour-joining tree made from all SNPs called relative to the SG-M1 reference, a set of strains containing all CC283 strains and strains from the nearest non-CC283 clade (consisting of 92 strains within CC10) was taken. We excluded those strains which had no metadata for isolation date, leaving a total of 273 strains (215 from the ST283 clade, 58 from the non-ST283 clade). We reconstructed an aligned genome sequence for each of these 273 strains by introducing that strain’s SNPs into the SG-M1 genome; gaps were also inserted where mapping coverage was below 10 as per Lofreq default parameters. The BRATNextGen [19] (using a clustering cut off of 0.2 and a significance cut off of 0.05) and ClonalFrameML [20] (using default parameters) programs were used to call recombination on this set of 273 aligned genomes. We took the union of all recombined segments called by either program in any of the 273 strains as the maximal recombination set (in total, 608 kb or 28.7% of the total chromosome); these were removed for the subsequent Bayesian analysis. We also took the union of all recombined segments called by either program only in the 215 CC283 strains as a CC283-specific recombination set (totalling 62 kb or 3.0% of the total chromosome); removal of these segments from the reconstructed genomes of the original 227 CC283 strains (including those that had no available year or country metadata) resulted in a recombination-free CC283 alignment which was used to generate the maximum-likelihood tree shown in Fig 2B.

#### Phylogenetic trees

Approximately maximum-likelihood SNP trees were created using FastTree 2.1.8 with the–gtr and–nt command line options [21] on reconstructed genome sequences (relative to the SG-M1 reference genome). Fig 2A was constructed from 1,236 strains (all CC10 and CC283 strains; all strains described in Da Cunha, et al 2014 [22] that were available in GenBank; and 23 complete genomes in the GenBank Refseq database as of August 4, 2016) using all called SNPs (i.e., no removal of recombination). Fig 2B was created from all CC283 strains after removal of CC283-specific recombination as described above (Recombination analysis). All phylogenetic trees were visualised with GGTREE 3.2 [23] in R (3.2.2) (https://www.R-project.org). Origins of the GBS CC283 WGS sequences used for phylogenetic analysis are given in Table 1.

**Table 1:** New and Existing group B *Streptococcus* (GBS) clonal complex (CC) 283 sequences used for phylogenetic analysis. This gives an overview of the origins and numbers of CC283. Further details are in Supporting Information.

Host	Country	Site or reference	Collection Year	Sample type	GBS isolates screened for ST283 (No.)	GBS isolates with WGS data (No.)	CC283 isolates with WGS data (No.)
**New sequences**							
Human	Singapore	Singapore General Hospital	1998	B	5	5	5
Human	Singapore	Tan Tock Seng Hospital & Changi General Hospital	2001–2010	B, CSF	331	331	21
Human	Thailand	Nakhon Phanom and Sa Kaeo provinces	2007–2015	B	139	22 ^a^	22
Human	Lao PDR	Mahosot Hospital, Vientiane	2000–2017	B, CSF	38	38	30^b^
Human	Vietnam	Hospital for Tropical Diseases, Ho Chi Minh City	2015–2017	B	13	13	4
Human	Vietnam	Phu San Hospital, Hanoi	2016–2017	HVS	38	0 ^a^	0
Human	Cambodia	Angkor Hospital for Children, Siem Reap	2012–2016	S	11	0 ^a^	0
Human	Britain	Multiple hospitals	2015	B	ND	1,017	1
Tilapia	Malaysia	Kedah and Terengganu states	2007–2008	Br, E, K	28	28	28
Tilapia	Vietnam	An Giang and Can Tho	2016	Br	34	34	34
**Existing sequences**							
Human	Singapore	[1]	2011–2015	B, CSF	ND	145	40
Human	Thailand	[1]	2015	B	ND	7	6
Human	Hong Kong	[24]	1993–2012	SS	ND	11	11
Human	Netherlands	GenBank	ND	ND	ND	1	1
Fish	Singapore	[1, 10]	2015	S, O, M	ND	20	20
Fish	Thailand	[9]	2000–2010	ND	ND	1	1
Fish	Vietnam	[9]	2006	ND	ND	1	1
Fish	Thailand	[25]	2012–2014	ND	ND	1	1
Frog	China	GenBank	2014	L	ND	1	1

^a^ = WGS was only performed on GBS positive by an ST283 specific PCR.

^b^ = Included one technical replicate. Where sequences from the same site were identical (based on SNP calls), only one was used. Abbreviations: No. = number, B = blood, CSF = cerebro-spinal fluid, HVS = high vaginal swab, S = superficial swabs, ND = not determined, SS = sterile site, Br = brain, E = eye, K = kidney, O = organs, M = muscle, L = liver.

#### BEAST analysis

This was performed on all CC10 and CC283 strains described above in the recombination section. Analysis of divergence times was performed using BEAST 2.4.8 [26] using a combination of site substitution models (HKY, GTR4), clock models (strict, relaxed exponential, relaxed lognormal), and population change models (coalescent constant, exponential, Bayesian skyline, and extended Bayesian skyline). MCMC runs were done in triplicate with 200,000,000 steps, using the first 20,000,000 as burn-in, sampling every 10,000 steps. Logcombiner was used to combine the three runs. Models were compared using AICM analysis in Tracer V1.6 [27], from which the GTR4 substitution model with a strict clock and exponential model was chosen as the best set of parameters. Treeannotator was used to generate a final tree. Visualization was done in FigTree 1.4.3 (http://tree.bio.ed.ac.uk/software/figtree/). The divergence time reported was calculated after removing the maximal recombination set; since this was a significant fraction of the genome, we repeated the analysis using a data set from which only the ST283-specific recombination regions were removed. The 95% highest posterior density for the maximal recombination data set fell entirely within that for the ST283-specific recombination set, suggesting that the removal of additional recombination regions did not drastically bias this result. However, despite this technical agreement, the opportunistic nature of the source data for these predictions must be kept in mind when interpreting these results.

## Results

### Human GBS CC283 in Asia

GBS ST283 accounted for 11% to 76% of invasive GBS per country, with the earliest known example collected in 1995, in Hong Kong. Five GBS ST11, single locus variants (SLV) of ST283, isolated from meningitis cases in Singapore in 1998 [28, 29] were recently corrected to ST283 [30], leaving no known examples of ST11. In total, 29% of GBS were identified as ST283 (Fig 1, Table 2). The majority of patients with ST283 (345/357 (97%) were adults, and 36% to 80% did not have comorbidities. Meningitis, endocarditis, and septic arthritis were noted in 10% to 35%, 4.5% to 10%, and 23% to 39% of adult patients with ST283, respectively (S1 Table).

**Table 2:** Asian^a^ Human group B *Streptococcus* (GBS) clonal complex (CC) 283 reported up to December 2017 as a proportion of invasive GBS by location and year. All CC283 in this table are sequence type (ST) 283: other examples of CC283 were not found. This table shows that ST283 was found in the first year of all newly described GBS collections in Southeast Asia, so it may have predated these collections, and that both GBS numbers available and ST283 proportions vary from year to year.

	Vientiane, Lao PDR		Nakhon Phanom and Sa Kaeo provinces, Thailand		Ho Chi Minh City, Vietnam		Singapore ^b^		Total	
Year	GBS No.	CC283 No. (%)	GBS No.	CC283 No. (%)	GBS No.	CC283 No. (%)	GBS No.	CC283 No. %	GBS No.	CC283 No. (%)
2000	1	1 (100)	ND	ND	ND	ND	ND	ND	1	1
2001	0	0	ND	ND	ND	ND	16	3 (19)	16	3 (19)
2002	0	0	ND	ND	ND	ND	13	2 (15)	13	2 (15)
2003	1	1 (100)	ND	ND	ND	ND	28	2 (7)	29	3 (10)
2004	0	0	ND	ND	ND	ND	32	7 (22)	32	7 (22)
2005	0	0	ND	ND	ND	ND	23	0 (0)	23	0 (0)
2006	3	2 (67)	ND	ND	ND	ND	36	2 (6)	38	4 (11)
2007	5	5 (100)	7	3 (43)	ND	ND	41	2 (5)	52	10 (19)
2008	0	0	10	6 (60)	ND	ND	46	1 (2)	54	7 (13)
2009	2	1 (50)	1	0	ND	ND	53	1 (2)	55	2 (4)
2010	7	5 (71)	4	3 (75)	ND	ND	43	1 (2)	52	9 (17)
2011	1	0 (0)	5	4 (80)	ND	ND	38	0 (0)	44	4 (9)
2012	1	1 (100)	13	8 (62)	ND	ND	43	3 (7)	57	12 (21)
2013	3	3 (100)	31	27 (87)	ND	ND	58	9 (16)	92	39 (42)
2014	2	1 (50)	66	50 (76)	ND	ND	77	15 (19)	145	66 (46)
2015	4	3 (75)	2	1 (50)	4	1 (25)	110	61 (55)*	120	66 (55)
2016	7	5 (71)	ND	ND	8	3 (38)	50	15 (30)	15	8 (53)
2017	1	1 (100)	ND	ND	1	0 (0)	ND	ND	2	1 (50)
Total	38	29 (76)	139	102 (73)	13	4 (31)	707	124 (18)	840	244 (29)

^a^ Similarly detailed data for Hong Kong were not found.

^b^ This Singapore data is from the only two institutions that had systematically saved GBS; Tan Tock Seng Hospital and Changi General Hospital.

*The outbreak year. Abbreviations: ND = not determined.

### Piscine GBS CC283 in Asia

All newly sequenced GBS isolated from tilapia in Peninsular Malaysia and Vietnam, during outbreaks of streptococcosis, were CC283 (Fig 1, Table 3). Notably, ten of the 28 fish sampled in Malaysia did not have signs of disease. ST283 was previously reported from 17/57 (30%) GBS isolated from seven freshwater fish species, including tilapia, on sale for human consumption in Singapore in 2015: these 17 ST283 were found in only 6/586 (1%) fish from ports, but in 11/39 (28%) fish from markets [10]. Two previously reported ST283 were reported from farmed tilapia in Thailand between 2000 and 2014 [9, 25]. One ST491 and one ST1311, (a new SLV of ST491, and also a double locus variant of ST283) were isolated from tilapia in Vietnam in 2006 [9] and 2016 respectively. An ST739, another SLV of ST283, was isolated in 2014 from a diseased tiger frog (*Hoplobatrachus chinensis*) farmed for human consumption in Guangdong, China, about 100Km from Hong Kong; although frogs are not piscine, they are often farmed in close proximity to fish and all known GBS infections in frogs are caused by clades that are also found in fishes [9].

**Table 3:** Asian Piscine group B *Streptococcus* (GBS) clonal complex (CC) 283 reported up to December 2017. All these CC283 were ST283, except for two variants, as indicated.

Country/fish	Data source	Date range	Locations	Sample type	GBS No.	CC283 No. (%)
Singapore; Asian bighead carp, red tilapia, black tilapia, giant snakehead, common snakehead, grass carp and silver carp ^a^	[10]	2015	Ports	I, S	27	6 (22)
			Markets	I, S	30	11 (36)
Peninsular Malaysia; red hybrid tilapia	New data	2007–2008	9 farms	I	28	28 (100)
Vietnam, tilapia	[9]	2006	ND	ND	ND	1 ^b^ (ND)
Vietnam, Can Tho and An Giang; tilapia	New data	2016	5 farms	I	34	34 ^**c**^ (100)
Thailand; tilapia	[9]	2000–2010	ND	I	8	1 (12·5)
Thailand; tilapia	[25]	2012–2014	ND	ND	ND	1 (ND)

^a^ A glossary of fish scientific names is in S2 Table.

^b^ This is ST491, a single locus variant of ST283.

^C^ One of these 34 is ST1311, a double locus variant of ST283 (See S3 Table). Abbreviations: I = invasive (brain, organs, muscle), S = superficial, ND = not determined.

### GBS CC283 outside Asia

Only four CC283, of human origin, were found: two from France, one from Britain, and one from the Netherlands (Fig 1, Table 4). All four were ST283 and none had associated epidemiological data. CC283 was otherwise absent from studies of over 4,000 human GBS from nine countries, of over 1,200 bovine GBS from five continents, and from the aquaculture literature from 6 continents, as of December 2017 (Fig 1 and S4 Table) [9, 22, 31–54]. Three human ST751, although an SLV of ST283 and ST10, were distinguished from ST283 by over 2,000 SNPs, based on phylogenetic analysis, and clustered outside of CC283, in CC10 (Fig 2), so ST751 is excluded from tables and figures referring to CC283. Other SLVs of ST283 listed in the MLST database are ST690, a human isolate, and ST160, of unknown origin, but we could not find WGS data for them. Three GBS serotype III-4 were not included as ST data were not available, although ST283 is the only published ST within serotype III-4: they were reported, without epidemiological data, amongst a collection from Australia and New Zealand in the original description of this subtype [55].

**Table 4:** Human group B *Streptococcus* (GBS) clonal complex (CC) 283 outside Asia.

ST, place/country, isolate No.	Data source	Date range	Sample type	GBS No.	ST283 No.	ST283%
ST283, Britain; ERR1742070	GenBank ^**a**^	2014	Invasive	1017	1	<0·1
ST283, France; S80 & S81	[8]	2002–2007	Invasive	119	2	2
ST283, Netherlands; ERR1659855	GenBank	ND	ND	ND	1	ND

^**a**^ Originally Public Health England (V. Chalker, personal communication). Abbreviations: ST = sequence type, ND = not determined.

### Genomic analysis

A previous report, using Bayesian analysis of a smaller subset of mostly Singaporean isolates, estimated the time of emergence of CC283 as 1994 (95% highest posterior density (HPD) 1991–1997) [1]. Analysis of our expanded set confirms that CC283 forms a monophyletic clade that appears to have arisen from within CC10 (Fig 2A). Bayesian analysis of our current set of CC283 and closely related CC10 strains suggests a slightly earlier predicted emergence date of 1985 (95% HPD 1980–1990). Given that the original Bayesian analysis was done on a data set that consisted largely of strains isolated from Singapore during 2015, the shift in the predicted emergence date is perhaps not surprising. However, our current data set was also not systematically collected, either by time, geography, or host, and these results should be interpreted in this context. Additional systematic sampling may provide further insights into emergence dates as well as the likely geographical and host origin of CC283. We found very little recombination in CC283 isolates, 62 kb (3.0%) of the total chromosome, and little variation in genome content, as indicated in the phylogenetic tree (Fig 2) and single nucleotide polymorphism (SNP) distances between CC283 pairs (S5 Table).

## Discussion

GBS CC283 has been widespread in SE Asia for over two decades in humans, and at least a decade in aquatic animals, but is rare outside SE Asia. As ST283 was found in the first year of every available collection of invasive GBS from SE Asia (Lao PDR, Thailand, Vietnam and Singapore), it may have been present even earlier. The estimated date of emergence, of 1985, predates the first known human cases and is roughly contemporaneous with the start of the expansion of aquaculture in SE Asia, based on reports from the Food and Agricultural Organisation of the United Nations [56]. The 2015 human outbreak of GBS in Singapore was fish borne; although our current study did not address transmission or consumption patterns, human cases in other parts of Southeast Asia could potentially also be fish-borne, especially since consumption of undercooked aquaculture foods is common in Asia, as shown by the high rate of trematode infections [57, 58]. It is not known to what extent healthy fish may carry ST283, or what the infectious dose for humans may be, but ST283 was isolated from healthy looking fish in Malaysian farms and in Singapore ports and markets, so fish sold for human consumption could be the source of exposure even if visibly diseased fish were excluded from sale. The high proportions of ST283 in invasive human GBS collections suggest that if human ST283 is acquired from aquaculture, or another undetermined food source, then invasive GBS is primarily a foodborne infection in Thailand and Lao PDR, and largely foodborne in Vietnam. The invasive human data from these three countries are all from areas bordering the Mekong River, separated by up to 1,500 km. If the ST283 data is not representative of each whole country, it may represent an epidemiology peculiar to the ecosystem surrounding the river.

### Aquaculture and one health

The phylogenetic tree shows that where human and fish GBS were collected concurrently, they are intermingled, whereas separate clustering of isolates of human and piscine origin tends to reflect collections being from different countries or time periods. We found numerous examples of pairs of human and piscine GBS distinguished by few to zero SNPs. Of note, while strict use of SNP distances is not fully reliable for determination of transmission, many of the examples have SNP distances smaller than 21 and are monophyletic in the ST283 tree. These criteria both support a possible “match”, as recently defined [59], although any SNP number cutoff is arbitrary, and the fewer the SNPs, the more likely is the linkage. GBS from the Singapore outbreak cluster with GBS from multiple countries, suggesting multiple sources contributed to the outbreak; although this may not seem surprising, given that Singapore imports fish from multiple countries, it leaves an unanswered question as to why imports from across the region suddenly, and simultaneously, were associated with increased GBS incidence in 2015. Of note, the 2015/2016 El Niño broke warming records in the central Pacific [60], and higher temperatures are associated with increased GBS load [61] and outbreaks of streptococcosis [62] in fish, and increased human cases [24].

GBS is known to affect fish species other than tilapia, including farmed and wild freshwater and marine fishes [63, 64]. Our detection of CC283 in fish farms was from tilapia, which were preferentially sampled because they are the most commercially important species affected by GBS in SE Asia, but the involvement of other species remains uncertain. Although the outbreak in Singapore was linked to several freshwater fish species, it is unclear whether the fish left the farms as carriers, or were contaminated during transport and handling. Cross contamination and post-contamination bacterial amplification at ambient temperature might explain the data, from Singapore, that showed that ST283 was recovered from 1% of fish taken from ports, but 28% of fish from markets [10].

Fish account for up to 37% of protein consumed in SE Asia [65], and Lao PDR and Thailand are among the top ten global tilapia producers [56]. Tilapia were introduced to SE Asia in the 1940s with repeated importations of fish fry: if these fry were contaminated at source, this could explain the finding of ST283 across the region. Alternatively, ST283 may have evolved regionally, from other animals, and acquired the ability to infect fishes through lateral gene transfer of virulence elements from other piscine GBS, followed by onward transmission [66]. Interestingly, GBS transmission from a tilapia hatchery into a new farm was reported in Malaysia where, in an effort to prevent economic losses due to streptococcosis, farms were advised to source fry from disease-free hatcheries [67]; GBS from these outbreaks in 2007/2008 were included in our study and are ST283. However, a survey of invasive GBS isolated from 13 adults in Malaysia in 2010 did not find Serotype III GBS [68]; this was a small sample and the predominant Malay population do not habitually eat raw fish, perhaps explaining this lack of human ST283.

There are a limited number of GBS clades that cause streptococcosis in fish, and ST283 and its variants are the only known serotype III GBS that naturally affect tilapia [9]. This suggests that ST283 caused streptococcosis in multiple sites across Thailand between 2003/2011, when 12% to 56% of GBS isolated from diseased tilapia were serotype III [69–71]; furthermore, some were serotype III-4, and the only described example of serotype III-4 is ST283 [7]. Interestingly, untyped GBS were reported as an emerging cause of septic arthritis, in humans, in Thailand between 1990 and 2010 [72].

The three predominant clades of GBS in fish are associated with different serotypes: ST7 with serotype Ia, CC552 (including ST260) with serotype Ib, and ST283 with serotype III [9]. Commercially available vaccines cover serotype Ib only, or serotypes Ia and III, but without cross-protection between serotypes. Strain confirmation is therefore recommended prior to use of vaccination, but diagnostic infrastructure is very limited in SE Asia, so fish farmers rely on antimicrobial treatment rather than on strain typing and vaccination. Antibiotics, including tetracyclines, are commonly used in controlling streptococcosis [73]. Concentrations above maximum limits have been found in fish sold for consumption in Vietnam [74], which may explain the high prevalence of tetracycline resistance genes found in ST283 from Vietnam. However, although similar antimicrobial use is reported in Thailand [75], tetracycline resistance amongst human ST283 from Thailand disappeared after 2012: this loss was also seen in human ST283 from Lao PDR and Singapore, possibly through three separate resistance gene loss events (Fig 2). In contrast, tetracycline resistance was 88% amongst 712 non-ST283 invasive human GBS, isolated in Singapore from 2001 to 2018 (T. Barkham, personal communication).

### Potential global dissemination

Whereas fish-associated CC7 and CC552 are geographically widespread, our literature review, up to December 2017, showed that CC283 had only been confirmed from SE Asia, although serotype III GBS had been described as the cause of disease outbreaks that occurred in 2016 in tilapia farms in Brazil [76]. Serotype III GBS isolates from Brazilian tilapia farms have now been confirmed as ST283, while this paper was in revision, and cluster with ST283 from Asia [25, 77]. This observation, in combination with import records of live Nile tilapia from Singapore to Brazil in 2014, suggests that ST283 may have been introduced into South America from Southeast Asia, underlining the potential threat of Southeast Asian ST283 to expanding aquaculture worldwide. The global trade in tilapia has previously been reported to account for the dissemination of GBS CC552 (ST260/261), from Israel to Australia, Africa (Ghana), Asia (China) and America (USA) [78] but CC552 does not affect homeothermic species. If ST283 spreads in a similar manner, human disease may also occur beyond SE Asia.

### Limitations and further studies

Our data is limited by the use of existing GBS collections. More extensive and systematic geographical sampling and transmission studies are needed. Future studies might specifically address the prevalence of GBS in fish hatcheries, animal feed, humans (including healthy carriers), and both healthy and diseased aquatic and non-aquatic animals, as well as the evolutionary origin and virulence mechanisms of ST283. Pathogenicity factors that explain the virulence of ST283 in humans are yet to be described, although a recent report identified the *bceR* gene as important for antibiotic resistance, biofilm formation, and lethality in a mouse model of infection [79]. Humans have previously been implicated as the source of GBS that caused a mass die-off of fish [80], so the interplay between aquaculture and human waste, which is commonly discharged into fish ponds and rivers in Asia, in part to recycle nutrients in the food production system, might also be studied.

### Summary

GBS ST283 is widespread in Southeast Asia, where it has been causing disease for over 25 years. Human ST283 is almost absent outside Asia but accounts for over 70% of invasive human GBS in collections from Thailand and Lao PDR. As both are significant producers of tilapia, and consumption of raw fish is common in these countries, we hypothesise that their ST283 infections are acquired from fish, as in the Singapore outbreak. ST283 has been detected in healthy and diseased farmed fish in SE Asia and is estimated to have emerged in 1985, corresponding with the beginning of the rapid expansion of the aquaculture industry. ST283 causing tilapia deaths in Brazil since 2016 are thought to have been introduced with tilapia imported from Asia in 2014. Evolutionary origins and routes of transmission within and between host species need further study, as it is possible that ST283 is transmitted from humans to aquaculture, or that there is another common source. Creation of cross-border collaborations in human and animal health are needed to complete the epidemiological picture, may lead to improved human and fish welfare, and may contribute to safer economic development in affected countries.

### Disclaimer

The findings and conclusions expressed by authors contributing in this manuscript do not necessarily reflect the official position of the Centers for Disease Control and Prevention or the institutions with which the authors are affiliated.

## Supporting information

S1 Table Human group B *Streptococcus* (GBS) sequence type (ST) ST283 in Asia: demographics and data sources. This table shows details for each human GBS collection, showing proportion of ST283, sample origins, dates and types, and age breakdown, foci of infection and gender.
(DOCX)

S2 Table Glossary of scientific names of fish. (DOCX)

S3 Table Details of known single locus variants, and one double locus variant, of group B *Streptococcus* sequence type (ST) 283. (DOCX)

S4 Table Global studies reporting group B streptococcus (GBS) Multi Locus Sequence Typing data, in which clonal complex (CC) 283 was not found. The absence of CC283 in this data, of over 4,000 human and over 1,300 animal GBS, demonstrate how unusual CC283 is. The data also show that Asia is not well represented in this group, with only one study found, from China.
(DOCX)

S5 Table Single Nucleotide Polymorphism (SNP) distances between pairs of group B Streptococcus clonal complex (CC) 283, from humans and animals. Examples were selected to show minimum and maximum SNP differences found between pairs within and between countries and hosts.
(DOCX)
