# Supplementary material for: One hypervirulent clone, sequence type 283, accounts for a large proportion of invasive Streptococcus agalactiae isolated from humans and diseased tilapia in Southeast Asia
Source: PLoS Negl Trop Dis. 2019 Jun 27;13(6):e0007421. doi: 10.1371/journal.pntd.0007421 (PMC6597049; doi:10.1371/journal.pntd.0007421)
Supplement: S1 Table — This table shows details for each human GBS collection, showing proportion of ST283, sample origins, dates and types, and age breakdown, foci of infection and gender. (DOCX) [file pntd.0007421.s001.docx]

**One hypervirulent clone, Sequence Type 283, accounts for a large proportion of invasive *Streptococcus agalactiae* isolated from humans and diseased tilapia in Southeast Asia.**

**S1 Table. Human group B *Streptococcus*(GBS) sequence type (ST) 283 in Asia: demographics and data sources.** This table shows details for each human GBS collection, showing proportion of ST283, sample origins, dates and types, and age breakdown, foci of infection and gender.

| **Place /country** | **Data**  **source** | **Date**  **range** | **No.**  **sites** | **Sample**  **type** | **GBS**  **No.** | **ST283**  **No. (%)** | **Neonates** | | **1 month**  **to 17 yrs** | **Adult ST283** | | |  | **All ages ST283** | | |  | **Age range (yrs) for ST238 isolates** | | | | | | |  | **Age range (yrs) for all GBS isolates** | | | | | | |  | **M:F ratio** |
| --- | --- | --- | --- | --- | --- | --- | --- | --- | --- | --- | --- | --- | --- | --- | --- | --- | --- | --- | --- | --- | --- | --- | --- | --- | --- | --- | --- | --- | --- | --- | --- | --- | --- | --- |
|  |  |  |  |  |  |  | **GBS**  **No.** | **ST283**  **No. (%)** | **ST283**  **No.** | **No.** | **Comor**  **%** | **M**  **No. (%)** |  | **OA**  **%** | **IE**  **%** | **Mor**  **%** |  | **<1** | **1-10** | **11-30** | **31-50** | **51-70** | **>70** | **ND** |  | **<1** | **1-10** | **11-30** | **31-50** | **51-70** | **>70** | **ND** |  |  |
| Singapore ^a^ | [1, 2] | 1998 | 1 | B | 5 | 5 (100) | 0 | 0 | 0 | 5 | 0 | 5 (100) |  | 0 | 0 | ND |  | 0 | 0 | 3 | 0 | 3 | 0 | 0 |  | ND | ND | ND | ND | ND | ND | ND |  | 50% male |
| Singapore ^b^ | [3] | 2011-2015 | 3 | B, C | 408 | 146 (36) | ND | ND | 0 | 146 | 20-40 | 29 (20) |  | 39 | 10 | 3·4 |  | For ST283 Median (IQR) age, yrs: 61.0 (50.0-68.0) | | | | | | |  | For non ST283Median (IQR) age, yrs: 69.5 (57.3–78.0) | | | | | | |  | 56% male |
| Singapore (new) | New data | 2001-2010 | 2 | B, C | 331 | 21 (6) | 0 | 0 | 0 | 21 | ND | ND |  | ND | ND | ND |  | 0 | 0 | 1 | 3 | 5 | 3 | 9 |  | 0 | 0 | 5 | 40 | 98 | 107 | 81 |  | ND |
| Hong Kong | [4] | 1993-2012 | 1 | I | 437 | 50 (11) | 113 | 5 (4) | 0 | 45 | 64 | 7 (16) |  | 23 | 4·5 | 27·3 |  | Mean age 63yrs (range 23-96) | | | | | | |  | ND | ND | ND | ND | ND | ND | ND |  | 50% male |
| Thailand: Nakhon Phanom and Sa Kaeo provinces | New data | 2007-2015 | 16 | B | 139 | 102 (73) | 6 | 0 | 1 | 101 | ND | 10 (10) |  | 10 | 0 | ND |  | 0 | 0 | 4 | 29 | 49 | 20 | 0 |  | 6 | 0 | 7 | 37 | 60 | 29 | 0 |  | ND |
| Vientiane, Lao PDR | New data | 2000-2017 | 1 | B, C | 38 | 29 (76) | 6 | 3 (50) | 3 | 23 | 34 | 8 (35) |  | ND | ND | ND |  | 4 | 0 | 7 | 7 | 7 | 4 | 0 |  | 7 | 0 | 7 | 9 | 10 | 5 | 0 |  | 66% male |
| Ho Chi Minh City Vietnam | New data | 2015-2017 | 1 | B | 13 | 4 (31) | 0 | 0 | 0 | 4 | ND | 1 (25) |  | ND | ND | ND |  | 0 | 0 | 1 | 1 | 2 | 0 | 0 |  | 0 | 0 | 2 | 3 | 6 | 2 | 0 |  | 53% male |
| Hanoi, Vietnam | New data | 2016-2017 | 1 | HVS | 38 | 0 | 0 | 0 | ND | ND | ND | ND |  | ND | ND | ND |  | ND | ND | ND | ND | ND | ND | ND |  | ND | ND | ND | ND | ND | ND | ND |  | ND |
| Siem Reap, Cambodia | New data | 2012-2016 | 1 | S | 11 | 0 | 5 | 0 | ND | ND | ND | ND |  | ND | ND | ND |  | 0 | 0 | 0 | 0 | 0 | 0 | 0 |  | 6 | 1 | 3 | 0 | 0 | 0 | 1 |  | 50% male |

^a^ refers to five ST283 previously incorrectly designated ST11, from a report of six GBS meningitis cases. ^b^ This report from Singapore includes seven ST283 from Bangkok, Thailand. Abbreviations: ND = not determined, B = blood, C = cerebro-spinal fluid, I = invasive, S = superficial, HVS = high vaginal swab. Comor = co-morbidities, M = meningitis, OA = osteoarticular & septic arthritis, IE = infective endocarditis, Mor = mortality.

1. Barkham T, Sheppard A, Jones N, Chen S. Streptococcus agalactiae that caused meningitis in healthy adults in 1998 are ST283, the same type that caused a foodborne outbreak of invasive sepsis in 2015: an observational molecular epidemiology study. Clin Microbiol Infect. 2018. Epub 2018/04/16. doi: 10.1016/j.cmi.2018.04.006. PubMed PMID: 29655956.

2. Wilder-Smith E, Chow KM, Kay R, Ip M, Tee N. Group B streptococcal meningitis in adults: recent increase in Southeast Asia. Aust N Z J Med. 2000;30(4):462-5. Epub 2000/09/14. PubMed PMID: 10985511.

3. Kalimuddin S, Chen SL, Lim CTK, Koh TH, Tan TY, Kam M, et al. 2015 Epidemic of Severe Streptococcus agalactiae Sequence Type 283 Infections in Singapore Associated With the Consumption of Raw Freshwater Fish: A Detailed Analysis of Clinical, Epidemiological, and Bacterial Sequencing Data. Clin Infect Dis. 2017;64(suppl_2):S145-S52. Epub 2017/05/06. doi: 10.1093/cid/cix021. PubMed PMID: 28475781.

4. Ip M, Ang I, Fung K, Liyanapathirana V, Luo MJ, Lai R. Hypervirulent Clone of Group B Streptococcus Serotype III Sequence Type 283, Hong Kong, 1993-2012. Emerg Infect Dis. 2016;22(10):1800-3. Epub 2016/09/21. doi: 10.3201/eid2210.151436. PubMed PMID: 27648702; PubMed Central PMCID: PMCPMC5038432.
