# Supplementary material for: One hypervirulent clone, sequence type 283, accounts for a large proportion of invasive Streptococcus agalactiae isolated from humans and diseased tilapia in Southeast Asia
Source: PLoS Negl Trop Dis. 2019 Jun 27;13(6):e0007421. doi: 10.1371/journal.pntd.0007421 (PMC6597049; doi:10.1371/journal.pntd.0007421)
Supplement: S2 Table — (DOCX) [file pntd.0007421.s002.docx]

**One hypervirulent clone, Sequence Type 283, accounts for a large proportion of invasive *Streptococcus agalactiae* isolated from humans and diseased tilapia in Southeast Asia.**

**Supporting information.**

**S2 Table. Glossary of scientific names of fish.**

| **Glossary of proper fish names** |  |
| --- | --- |
| Asian bighead carp | *Hypophthalmichthys nobilis* (Richardson, 1845) |
| Red tilapia or red hybrid tilapia **^a^** | *Oreochromis* sp. |
| Black or Nile tilapia | *Oreochromis niloticus* (Linnaeus, 1758) |
| Giant snakehead | *Channa micropeltes* (Cuvier, 1831) |
| Common snakehead | *Channa striata* (Bloch, 1793) |
| Grass carp | *Ctenopharyngodon idella* (Valenciennes, 1844) |
| Silver carp | *Hypophthalmichthys molitrix* (Valenciennes, 1844) |

**^a^** there are many hybrids, created by crossing different species. In Malaysia, *Oreochromis niloticus* × *O.* *mossambicus* is common*.*
