# Supplementary material for: One hypervirulent clone, sequence type 283, accounts for a large proportion of invasive Streptococcus agalactiae isolated from humans and diseased tilapia in Southeast Asia
Source: PLoS Negl Trop Dis. 2019 Jun 27;13(6):e0007421. doi: 10.1371/journal.pntd.0007421 (PMC6597049; doi:10.1371/journal.pntd.0007421)
Supplement: S3 Table — (DOCX) [file pntd.0007421.s003.docx]

**One hypervirulent clone, Sequence Type 283, accounts for a large proportion of invasive *Streptococcus agalactiae* isolated from humans and diseased tilapia in Southeast Asia.**

**Supporting information.**

**Table S3. Details of known single locus variants, and one double locus variant, of group B *Streptococcus* sequence type (ST) 283.**

| **ST, isolate, country** | **Data source** | **Date** | **Source** | **Sample type** | **SLV or DLV** | **No.** | **WGS No.** |
| --- | --- | --- | --- | --- | --- | --- | --- |
| ST491; STIR-CD-14, Vietnam. | [1] | 2006 | Tilapia | ND | SLV | 1 | 1 |
| ST1311; MRI-Z2-398, Vietnam | New data | 2016 | Tilapia | Invasive | DLV | 1 | 1 |
| ST739; FWL1405, Guangdong, China | MLST database | 2014 | Frog | Invasive | SLV | 1 | 1 |
| ST160; no isolates found, ND | MLST database | ND | ND | ND | SLV | 0 | 0 |
| ST11; A1_211, A2_211, A4_211, A5_211, A6_211, Singapore | [2] | 1998 | Human | Blood | SLV | 0 **^a^** | 0 |
| ST690; A35 (serotype V), Algeria | MLST database | ND | Human | HVS | SLV | 1 | 0 ^b^ |
| ST751. ERR1625336, ERR1672888 (both from The Netherlands) & MRI-Z2-193 (Sweden) [3]. All are serotype II | GenBank | 2014 | Human | ND | SLV | 3 | 2 ^b^ |

^a^ All five ST11 were corrected to ST283. ^b^ WGS data not available for one isolate. Abbreviations: ST = sequence type, SLV = single locus variant, DLV = double locus variant, No. = Number of each sequence type reported, WGS No. = Number of whole genome sequences found, ND = not determined, HVS = high vaginal swab.

1. Delannoy CM, Crumlish M, Fontaine MC, Pollock J, Foster G, Dagleish MP, et al. Human Streptococcus agalactiae strains in aquatic mammals and fish. BMC Microbiol. 2013;13:41. Epub 2013/02/20. doi: 10.1186/1471-2180-13-41. PubMed PMID: 23419028; PubMed Central PMCID: PMCPMC3585737.

2. Barkham T, Sheppard A, Jones N, Chen S. Streptococcus agalactiae that caused meningitis in healthy adults in 1998 are ST283, the same type that caused a foodborne outbreak of invasive sepsis in 2015: an observational molecular epidemiology study. Clin Microbiol Infect. 2018. Epub 2018/04/16. doi: 10.1016/j.cmi.2018.04.006. PubMed PMID: 29655956.

3. Lyhs U, Kulkas L, Katholm J, Waller KP, Saha K, Tomusk RJ, et al. Streptococcus agalactiae Serotype IV in Humans and Cattle, Northern Europe. Emerg Infect Dis. 2016;22(12):2097-103. Epub 2016/11/22. doi: 10.3201/eid2212.151447. PubMed PMID: 27869599; PubMed Central PMCID: PMCPMC5189126.
