# Supplementary material for: One hypervirulent clone, sequence type 283, accounts for a large proportion of invasive Streptococcus agalactiae isolated from humans and diseased tilapia in Southeast Asia
Source: PLoS Negl Trop Dis. 2019 Jun 27;13(6):e0007421. doi: 10.1371/journal.pntd.0007421 (PMC6597049; doi:10.1371/journal.pntd.0007421)
Supplement: S4 Table — The absence of CC283 in this data, of over 4,000 human and over 1,300 animal GBS, demonstrate how unusual CC283 is. The data also show that Asia is not well represented in this group, with only one study found, from China. (DOCX) [file pntd.0007421.s004.docx]

**One hypervirulent clone, Sequence Type 283, accounts for a large proportion of invasive *Streptococcus agalactiae* isolated from humans and diseased tilapia in Southeast Asia.**

**Supporting information.**

**S4 Table. Global studies reporting group B *Streptococcus* (GBS) Multi Locus Sequence Typing data, in which clonal complex (CC) 283 was not found.** The absence of CC283 in this data, of over 4,000 human and over 1,300 animal GBS, demonstrate how unusual CC283 is. The data also show that Asia is not well represented in this group, with only one study found, from China.

| **Continent or Country** | **Years** | **No. GBS** | **Host** | **Disease or Colonization** |
| --- | --- | --- | --- | --- |
| Africa [1] | 2005-2006 | 163 | Human maternal | Colonisation |
| Africa [2] | 2007-2010 | 169 | Human adult | 95 vaginal colonisation  74 invasive |
| Australia [3] | 2008-2011 | 40 | Fish (several wild species) | Disease |
| Australia (a) | 2015 | 22 | Cattle | Mastitis (3 farms) |
| Brazil [4] | 2003-2015 | 39 | Catfish (n = 1)  Nile tilapia (n = 38) | Disease outbreaks on fish farms |
| Canada [5] | 1993-2002 | 192 & 232 | Human neonatal & young children | 192 invasive and 232 vaginal colonisation |
| Canada [6] | 2010-2014 | 85 | Human adult | Invasive |
| China [7] | 2011 | 102 | Cattle | Mastitis |
| Colombia [8] | 2003-2011 | 24 | Tilapia | Disease |
| Colombia [18] | 2009-2011 | 40 | Cattle | Mastitis |
| Colombia [9] | 2013-2014 | 207 | Cattle | Mastitis |
| Denmark [19] | 2009 | 111 | Cattle | Mastitis (national bulk tank milk surveillance) |
| Denmark [10] | 2011 | 71 | Cattle | Mastitis (6 herds) |
| Finland [20] | 2011-2012  2010-2012 | 69  63 | Humans  Cattle | All ages; UTI, SSTI, carriage  Mastitis (29 herds) |
| France [11] | 1996-1997 | 3 | Cattle | Mastitis |
| Ghana [3] | 2016 | 4 | Nile tilapia | Disease |
| Honduras [3] | 2014 | 9 | Nile tilapia | Disease |
| Iceland [12] | 1975-2014 | 145 | Human (adults) | Invasive disease |
| Italy (b) | 2003-2008 | 11 | Cattle | Mastitis |
| Norway [21] | 2013-2014 | 54 | Cattle | Mastitis, rectal swabs, vaginal swabs, farm environment (14 farms) |
| Poland [13] | 1996-2005 | 114 | Human all ages | Invasive and carriage |
| Portugal [14] | 2000-2004 | 75 | Human (neonates/pregnant women) | Invasive disease /colonization |
| Portugal [15] | 2001-2008 | 225 | Human (adults) | Invasive disease |
| Portugal [16] | 2005-2015 | 218 | Human (neonates) | Invasive disease |
| Portugal [17] | 2009-2015 | 555 | Human (adults) | Invasive disease |
| Portugal (c) | 2005-2015 | 318 | Human (adults) | Non-invasive disease |
| Portugal [11] | 2002-2003  2011-2014 | 17  197 | Cattle | Mastitis |
| Scotland (d) | 2001-2015 | 11 | Cattle | Mastitis (6 herds) |
| Spain [18] | 1992-2009 | 212 | Human (neonates) | Invasive disease |
| Spain [11] | 2005-2006 | 17 | Cattle | Mastitis |
| Sweden [19] | 1988-1997 | 158 | Human adult and neonate | Invasive disease |
| Sweden [20] | Ca. 2010-2012  2010-2012 | 12  45 | Human  Cattle | Invasive disease  Mastitis |
| United Kingdom [20] | 1987-1996 | 111 | Cattle | Mastitis |
| USA [21] | 1995-1999 | 899 | Human neonate | 129 invasive & 770 colonising |
| Multi-national from four continents [22] | 1953-2011 | 216 & 13 | Human & animal | Invasive and carriage |
| 9 countries from five continents [23] | Not stated | 128 & 139 | Human and Cattle | Human invasive and colonisers & cattle mastitis |
| Multinational (Brazil, Honduras, Israel, Kuwait, USA) [24] | 2001 (Kuwait); not stated | 21 | Fish (mullet, seabream, hybrid striped bass, Nile tilapia) | Disease |

1. Bosward K and Zadoks RN, (new data)
2. Moroni P and Zadoks RN, (new data)
3. Martins ER, (new data)
4. Zadoks RN, (new data)

1. Brochet M, Couve E, Bercion R, Sire JM, Glaser P. Population structure of human isolates of Streptococcus agalactiae from Dakar and Bangui. J Clin Microbiol. 2009;47(3):800-3. Epub 2008/12/26. doi: 10.1128/JCM.01103-08. PubMed PMID: 19109468; PubMed Central PMCID: PMCPMC2650903.

2. Huber CA, McOdimba F, Pflueger V, Daubenberger CA, Revathi G. Characterization of invasive and colonizing isolates of Streptococcus agalactiae in East African adults. J Clin Microbiol. 2011;49(10):3652-5. Epub 2011/08/26. doi: 10.1128/JCM.01288-11. PubMed PMID: 21865428; PubMed Central PMCID: PMCPMC3187314.

3. Kawasaki M, Delamare-Deboutteville J, Bowater RO, Walker MJ, Beatson S, Ben Zakour NL, et al. Microevolution of Streptococcus agalactiae ST-261 from Australia Indicates Dissemination via Imported Tilapia and Ongoing Adaptation to Marine Hosts or Environment. Appl Environ Microbiol. 2018;84(16). Epub 2018/06/20. doi: 10.1128/AEM.00859-18. PubMed PMID: 29915111; PubMed Central PMCID: PMCPMC6070751.

4. Barony GM, Tavares GC, Pereira FL, Carvalho AF, Dorella FA, Leal CAG, et al. Large-scale genomic analyses reveal the population structure and evolutionary trends of Streptococcus agalactiae strains in Brazilian fish farms. Sci Rep. 2017;7(1):13538. Epub 2017/10/21. doi: 10.1038/s41598-017-13228-z. PubMed PMID: 29051505; PubMed Central PMCID: PMCPMC5648781.

5. Manning SD, Springman AC, Lehotzky E, Lewis MA, Whittam TS, Davies HD. Multilocus sequence types associated with neonatal group B streptococcal sepsis and meningitis in Canada. J Clin Microbiol. 2009;47(4):1143-8. Epub 2009/01/23. doi: 10.1128/JCM.01424-08. PubMed PMID: 19158264; PubMed Central PMCID: PMCPMC2668308.

6. Teatero S, Athey TB, Van Caeseele P, Horsman G, Alexander DC, Melano RG, et al. Emergence of Serotype IV Group B Streptococcus Adult Invasive Disease in Manitoba and Saskatchewan, Canada, Is Driven by Clonal Sequence Type 459 Strains. J Clin Microbiol. 2015;53(9):2919-26. Epub 2015/07/03. doi: 10.1128/JCM.01128-15. PubMed PMID: 26135871; PubMed Central PMCID: PMCPMC4540936.

7. Yang Y, Liu Y, Ding Y, Yi L, Ma Z, Fan H, et al. Molecular characterization of Streptococcus agalactiae isolated from bovine mastitis in Eastern China. PLoS One. 2013;8(7):e67755. Epub 2013/07/23. doi: 10.1371/journal.pone.0067755. PubMed PMID: 23874442; PubMed Central PMCID: PMCPMC3707890.

8. Barato P, Martins ER, Melo-Cristino J, Iregui CA, Ramirez M. Persistence of a single clone of Streptococcus agalactiae causing disease in tilapia (Oreochromis sp.) cultured in Colombia over 8 years. J Fish Dis. 2015;38(12):1083-7. Epub 2015/02/04. doi: 10.1111/jfd.12337. PubMed PMID: 25643734.

9. Cobo-Angel C, Jaramillo-Jaramillo AS, Lasso-Rojas LM, Aguilar-Marin SB, Sanchez J, Rodriguez-Lecompte JC, et al. Streptococcus agalactiae is not always an obligate intramammary pathogen: Molecular epidemiology of GBS from milk, feces and environment in Colombian dairy herds. PLoS One. 2018;13(12):e0208990. Epub 2018/12/12. doi: 10.1371/journal.pone.0208990. PubMed PMID: 30532177; PubMed Central PMCID: PMCPMC6287850.

10. Mahmmod YS, Klaas IC, Katholm J, Lutton M, Zadoks RN. Molecular epidemiology and strain-specific characteristics of Streptococcus agalactiae at the herd and cow level. J Dairy Sci. 2015;98(10):6913-24. Epub 2015/08/04. doi: 10.3168/jds.2015-9397. PubMed PMID: 26233443.

11. Almeida A, Alves-Barroco C, Sauvage E, Bexiga R, Albuquerque P, Tavares F, et al. Persistence of a dominant bovine lineage of group B Streptococcus reveals genomic signatures of host adaptation. Environ Microbiol. 2016;18(11):4216-29. Epub 2016/10/26. doi: 10.1111/1462-2920.13550. PubMed PMID: 27696631.

12. Bjornsdottir ES, Martins ER, Erlendsdottir H, Haraldsson G, Melo-Cristino J, Kristinsson KG, et al. Changing epidemiology of group B streptococcal infections among adults in Iceland: 1975-2014. Clin Microbiol Infect. 2016;22(4):379 e9- e16. Epub 2015/12/23. doi: 10.1016/j.cmi.2015.11.020. PubMed PMID: 26691681.

13. Sadowy E, Matynia B, Hryniewicz W. Population structure, virulence factors and resistance determinants of invasive, non-invasive and colonizing Streptococcus agalactiae in Poland. J Antimicrob Chemother. 2010;65(9):1907-14. Epub 2010/06/30. doi: 10.1093/jac/dkq230. PubMed PMID: 20584746.

14. Martins ER, Pessanha MA, Ramirez M, Melo-Cristino J, Portuguese Group for the Study of Streptococcal I. Analysis of group B streptococcal isolates from infants and pregnant women in Portugal revealing two lineages with enhanced invasiveness. J Clin Microbiol. 2007;45(10):3224-9. Epub 2007/08/19. doi: 10.1128/JCM.01182-07. PubMed PMID: 17699641; PubMed Central PMCID: PMCPMC2045366.

15. Martins ER, Melo-Cristino J, Ramirez M, Portuguese Group for the Study of Streptococcal I. Dominance of serotype Ia among group B Streptococci causing invasive infections in nonpregnant adults in Portugal. J Clin Microbiol. 2012;50(4):1219-27. Epub 2012/01/06. doi: 10.1128/JCM.05488-11. PubMed PMID: 22219307; PubMed Central PMCID: PMCPMC3318525.

16. Martins ER, Pedroso-Roussado C, Melo-Cristino J, Ramirez M, Portuguese Group for the Study of Streptococcal I. Streptococcus agalactiae Causing Neonatal Infections in Portugal (2005-2015): Diversification and Emergence of a CC17/PI-2b Multidrug Resistant Sublineage. Front Microbiol. 2017;8:499. Epub 2017/04/13. doi: 10.3389/fmicb.2017.00499. PubMed PMID: 28400757; PubMed Central PMCID: PMCPMC5368217.

17. Lopes E, Fernandes T, Machado MP, Carrico JA, Melo-Cristino J, Ramirez M, et al. Increasing macrolide resistance among Streptococcus agalactiae causing invasive disease in non-pregnant adults was driven by a single capsular-transformed lineage, Portugal, 2009 to 2015. Euro Surveill. 2018;23(21). Epub 2018/05/31. doi: 10.2807/1560-7917.ES.2018.23.21.1700473. PubMed PMID: 29845930.

18. Martins ER, Andreu A, Correia P, Juncosa T, Bosch J, Ramirez M, et al. Group B streptococci causing neonatal infections in barcelona are a stable clonal population: 18-year surveillance. J Clin Microbiol. 2011;49(8):2911-8. Epub 2011/06/24. doi: 10.1128/JCM.00271-11. PubMed PMID: 21697333; PubMed Central PMCID: PMCPMC3147731.

19. Luan SL, Granlund M, Sellin M, Lagergard T, Spratt BG, Norgren M. Multilocus sequence typing of Swedish invasive group B streptococcus isolates indicates a neonatally associated genetic lineage and capsule switching. J Clin Microbiol. 2005;43(8):3727-33. Epub 2005/08/06. doi: 10.1128/JCM.43.8.3727-3733.2005. PubMed PMID: 16081902; PubMed Central PMCID: PMCPMC1233917.

20. Bisharat N, Crook DW, Leigh J, Harding RM, Ward PN, Coffey TJ, et al. Hyperinvasive neonatal group B streptococcus has arisen from a bovine ancestor. J Clin Microbiol. 2004;42(5):2161-7. Epub 2004/05/08. PubMed PMID: 15131184; PubMed Central PMCID: PMCPMC404684.

21. Bohnsack JF, Whiting A, Gottschalk M, Dunn DM, Weiss R, Azimi PH, et al. Population structure of invasive and colonizing strains of Streptococcus agalactiae from neonates of six U.S. Academic Centers from 1995 to 1999. J Clin Microbiol. 2008;46(4):1285-91. Epub 2008/02/22. doi: 10.1128/JCM.02105-07. PubMed PMID: 18287314; PubMed Central PMCID: PMCPMC2292926.

22. Da Cunha V, Davies MR, Douarre PE, Rosinski-Chupin I, Margarit I, Spinali S, et al. Streptococcus agalactiae clones infecting humans were selected and fixed through the extensive use of tetracycline. Nat Commun. 2014;5:4544. Epub 2014/08/05. doi: 10.1038/ncomms5544. PubMed PMID: 25088811; PubMed Central PMCID: PMCPMC4538795.

23. Sorensen UB, Poulsen K, Ghezzo C, Margarit I, Kilian M. Emergence and global dissemination of host-specific Streptococcus agalactiae clones. MBio. 2010;1(3). Epub 2010/09/09. doi: 10.1128/mBio.00178-10. PubMed PMID: 20824105; PubMed Central PMCID: PMCPMC2932510.

24. Evans JJ, Bohnsack JF, Klesius PH, Whiting AA, Garcia JC, Shoemaker CA, et al. Phylogenetic relationships among Streptococcus agalactiae isolated from piscine, dolphin, bovine and human sources: a dolphin and piscine lineage associated with a fish epidemic in Kuwait is also associated with human neonatal infections in Japan. J Med Microbiol. 2008;57(Pt 11):1369-76. Epub 2008/10/18. doi: 10.1099/jmm.0.47815-0. PubMed PMID: 18927414.
