# Supplementary material for: One hypervirulent clone, sequence type 283, accounts for a large proportion of invasive Streptococcus agalactiae isolated from humans and diseased tilapia in Southeast Asia
Source: PLoS Negl Trop Dis. 2019 Jun 27;13(6):e0007421. doi: 10.1371/journal.pntd.0007421 (PMC6597049; doi:10.1371/journal.pntd.0007421)
Supplement: S5 Table — Examples were selected to show minimum and maximum SNP differences found between pairs within and between countries and hosts. (DOCX) [file pntd.0007421.s005.docx]

**One hypervirulent clone, Sequence Type 283, accounts for a large proportion of invasive *Streptococcus agalactiae* isolated from humans and diseased tilapia in Southeast Asia.**

**Supporting information.**

**S5 Table. Single Nucleotide Polymorphism (SNP) distances between pairs of group B *Streptococcus* clonal complex (CC) 283, from humans and animals.** Examples were selected to show minimum and maximum SNP differences found between pairs within and between countries and hosts.

| **Country** | **Host** | **Year isolated** | **Difference in years** | **SNPs**  **Min no.** | **SNPs**  **Max no.** |
| --- | --- | --- | --- | --- | --- |
| Singapore  Singapore | H  H | 1998  2014 | 16 | 15 | - |
| Singapore  Singapore | H  H | 2012-2015  2015 | 0-3 | 0 | - |
| Singapore  Singapore | H  F | 2015  2015 | 0 | 0 | - |
| Singapore  Singapore | H  F | 2001  2015 | 14 | 30 | - |
| Singapore  Malaysia | H  F | 2003  2007 | 4 | 5 | - |
| Singapore  Malaysia | H  F | 2007  2007 | 0 | 6 | - |
| Singapore  Lao PDR | H  H | 2015  2015 | 0 | 2 | - |
| Singapore  Thailand | H  F | 2007  2012-2014 | 5-7 | 13 | - |
| Vietnam  Vietnam | H  F | 2015  2016 | 1 | 23 | - |
| Thailand  Thailand | F  H | 2012-2014  2015 | 1-3 | 8 | - |
| Thailand Lao PDR | H  H | 2007  2010 | 3 | 2 | - |
| Lao PDR  Lao PDR | H  H | 2007  2016 | 9 | 2 | - |
| Hong Kong Britain | H  H | 2008  2014 | 6 | 9 | - |
| Hong Kong  China | H  Fr | 1998  2014 | 16 | 41 |  |
| Hong Kong  Vietnam | H  H | 1998  2016 | 18 | - | 206 |
| Vietnam  Thailand | F  H | 2016  2015 | 1 | - | 183 |
| Lao PDR  Vietnam | H  H | 2000  2016 | 16 | - | 193 |
| Thailand  Vietnam | H  H | 2015  2016 | 1 | - | 357 |

Abbreviations: SNP = single nucleotide polymorphism H = human, F = fish, Fr = frog.
